# NO Emission Characteristics of Pulverized Coal Combustion in O_2_/N_2_ and O_2_/H_2_O Atmospheres in a Drop-Tube Furnace

**DOI:** 10.3390/ma17204997

**Published:** 2024-10-12

**Authors:** Liang Zhang, Jun Fan, Changlin Wang, Jiaqi Yuan, Cen Hao, Shiying Cao

**Affiliations:** 1 School of Information Engineering, Jiangsu Open University, Nanjing 210036, China; zhangliang@jsou.edu.cn (L.Z.); fanjun@jsou.edu.cn (J.F.); wangcl@jsou.edu.cn (C.W.); nayuanjiaqi@126.com (J.Y.); haocen@jsou.edu.cn (C.H.); 2 School of Mechanical and Electrical Engineering, Shenzhen Polytechnic University, Shenzhen 518055, China

**Keywords:** pulverized coal, O_2_/H_2_O atmosphere, drop-tube furnace, NO emissions

## Abstract

Oxy-steam combustion is a new oxy-fuel combustion technology. This paper focuses on the NO emission characteristics during the combustion of SF (Shen Fu) coal in O_2_/N_2_ and O_2_/H_2_O mixtures. Experiments were performed in a drop-tube furnace. Combustion tests were carried out in O_2_/N_2_ and O_2_/H_2_O atmospheres for various O_2_ concentrations (21%, 30%, 40%, and 60%) at different temperatures (1173 K, 1273 K, and 1373 K). In addition, combustion experiments at different excess oxygen ratios (λ) were conducted in O_2_/N_2_ and O_2_/H_2_O atmospheres. The influences of the atmosphere, oxygen concentration, temperature, and excess oxygen ratio on NO emissions were analyzed. The results show that the NO concentrations of SF coal combustion in the 21% O_2_/79% H_2_O atmosphere were much lower than those in the 21% O_2_/79% N_2_ atmosphere at the three temperatures considered. This was because a large amount of NO was decomposed during the SF coal combustion in the O_2_/H_2_O atmospheres. The reasons for the decomposition of NO include the selective non-catalytic reaction (SNCR) mechanism and char’s important role as a catalyst for the destruction of NO, either directly or by reacting with CO or H_2_. In oxy-steam combustion, the NO concentrations significantly increased with the increase in the oxygen concentration from 21 vol.% to 60 vol.% and the temperature from 1173 K to 1373 K. The excess oxygen ratio (λ) slightly impacted the NO emissions in the O_2_/H_2_O atmosphere.

## 1. Introduction

The energy produced by fossil fuel combustion results in carbon dioxide (CO_2_) emissions, the main reason for global warming; thus, reducing CO_2_ emissions is becoming increasingly important [1]. Carbon capture and storage (CCS) is an effective measure to control CO_2_ emissions. Oxy-fuel combustion, a promising CCS technology, has been a research hotspot of energy and combustion science in recent years due to its feasibility and low risk [2,3,4,5].

At present, oxy-fuel combustion technology is mainly represented by O_2_/CO_2_ recycle combustion. In O_2_/CO_2_ recycle combustion, fuels burn in mixtures of pure oxygen and recycled flue gas, resulting in a high concentration of CO_2_ in the flue gas, which is then ready for sequestration. The CO_2_ concentration in the flue gas is increased from approximately 17 to 70% by mass [6]. The advantages of O_2_/CO_2_ recycle combustion include higher coal reactivity, higher burnout rate, and lower NO_x_ emissions [3]; the main disadvantages are higher economic costs and lower efficiency than air-fired power plants [7].

The Canadian Centre for Mineral and Energy Technology (CANMET) developed a new-generation oxy-fuel system based on oxy-steam combustion in 2007. Water/steam is used to moderate the flame temperature in an oxy-steam combustion system. The recycling system is not mandatory. The exhaust gas mainly contains steam and CO_2_, which can be sent for compression and sequestration after H_2_O condensation [8]. The CANMET has developed a novel oxy-steam burner for zero-emission power plants. The computational fluid dynamics (CFD) simulation and pilot-scale experimental results showed that oxy-steam combustion led to high CO_2_ concentrations (−90%) and low CO, moderate NO_x_, and typical SO_x_ levels [9]. Seepana and Jayanti [10] proposed a power-generating system based on oxy-steam combustion called steam-moderated oxy-fuel combustion (SMOC). Compared with O_2_/CO_2_ recycle combustion, oxy-steam combustion has the following advantages [11]:

(1) The overall system is simple, compact, and easy to start up and shut down due to the little recycled flue gas.
(2) Oxy-steam combustion requires a much smaller amount of steam than O_2_/CO_2_ recycled combustion to achieve the same combustion temperature because of the significantly greater specific heat of steam compared with CO_2_. The system’s major and auxiliary equipment are smaller than those of the O_2_/CO_2_ recycled combustion system.
(3) The formation of NO_x_ and SO_x_ in the boiler can be reduced owing to the introduction of steam.
(4) The pumping costs associated with recycling are relatively low because the transmission medium is water and not flue gas.

The physical properties of H_2_O differ from those of N_2_ and CO_2_. Table 1 shows the physical properties of H_2_O, CO_2_, and N_2_. The heat capacity and mass diffusion coefficient of H_2_O are 1.26 and 1.34 times those of N_2_, respectively. Furthermore, the chemical properties of H_2_O are more active than those of N_2_ and CO_2_, such as its super-equilibrium radical effects and high chaperon efficiency [8]. Many researchers have investigated the effects of the physicochemical properties of H_2_O on the coal combustion process. The research results show that coal’s combustion characteristics and ignition mechanisms in the O_2_/H_2_O atmosphere differ from those in the O_2_/CO_2_ atmosphere [12,13,14,15,16,17,18].

The formation pathways of NO_x_ follow three routes during coal combustion: thermal and prompt formation from N_2_ and the oxidation of fuel-N. Thermal NO evolves from the reaction of N_2_ and O_2_ at high temperatures (above 1800 K), described by the extended Zeldovich mechanism. Prompt NO is of minor importance and occurs predominantly under fuel-rich conditions. Fuel NO is produced via the conversion of fuel-N (volatile-N and char-N) during coal combustion and contributes most of the total NO emissions [19,20].

The NO formation characteristics under an oxy-steam atmosphere are expected to differ from conventional air combustion and O_2_/CO_2_ combustion. Many researchers have investigated the effect of H_2_O on the NO_x_ precursor (NH_3_, HCN, and HCNO) formation during coal/biomass pyrolysis and gasification. The results show that NH_3_ is the major NO_x_ precursor of coal gasification in the presence of H_2_O, and the H radical is important for NH_3_ formation [21,22,23,24,25,26,27,28,29]. Park et al. [26,27] found that HCN was the primary product of the char-N reaction with H_2_O, and it depended on the availability of hydrogen on the char surface. Steam can improve the concentrations of free radicals (mainly H and OH), H_2_, and CO [30].

Although few studies have been conducted on the NO formation characteristics of coal combustion in an oxy-steam atmosphere, the impacts of H_2_O on the NO_x_ emissions in oxy-coal combustion under different conditions have been studied. The results indicate that NO_x_ emissions are reduced by adding H_2_O [31,32,33,34,35].

This paper focuses on the NO formation characteristics during the combustion of Shen Fu coal in O_2_/H_2_O mixtures. The experiments were performed in a drop-tube furnace (DTF). Two combustion atmospheres were considered: O_2_/H_2_O and O_2_/N_2_. The oxygen concentration varied between 21 vol.% and 60 vol.%. Furthermore, the effects of the temperature and excess oxygen ratio on the NO formation characteristics in the O_2_/H_2_O atmosphere were considered.

## 2. Materials and Methods

### 2.1. Coal Sample

Shen Fu (SF) bituminous coal was used in this study. The properties of the coal samples are shown in Table 2. The coal samples were crushed, ground, and sieved before use to obtain a particle size fraction of 88–97 μm.

### 2.2. Test Facility

**Figure 1:**
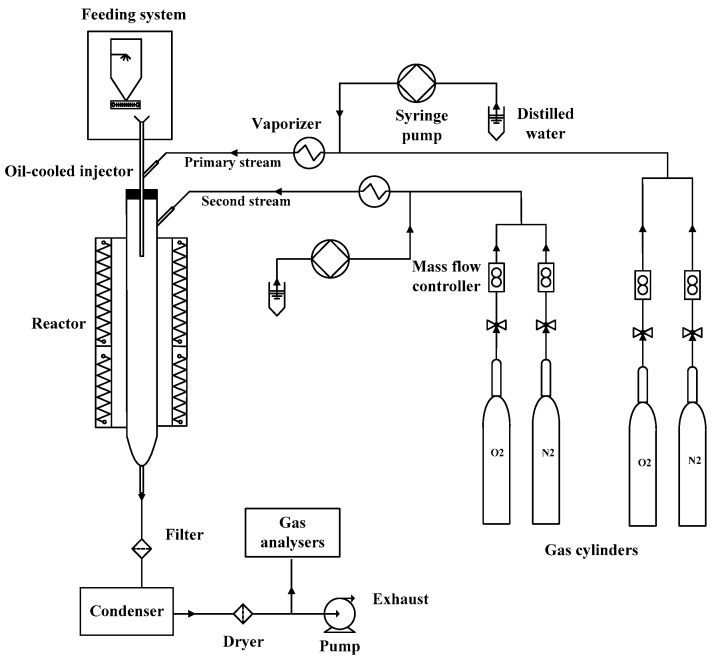
Schematic of the drop-tube furnace and auxiliary equipment.

The experiments were carried out in an electrically heated DTF, as shown in Figure 1. The furnace was a cylindrical quartz tube with a total length of 1900 mm and an inner diameter of 30 mm. The heating zone was 1360 mm long. The DTF could work at a maximum temperature of 1373 K, and the furnace wall temperatures were continuously monitored with type-K thermocouples embedded in the wall.

The coal samples were fed using a micro-feeding device and introduced via an oil-cooled injector to ensure the temperature did not exceed 423 K before entering the reaction zone. The reaction stream for combustion was composed of primary and secondary streams. The flow rates of O_2_ and N_2_ from the gas cylinders were controlled with mass flow controllers. A vaporizer generated the steam, in which distilled water and the inlet gas were heated up to 423 K. A high-precision syringe pump was used to control the flow rate of the distilled water. The inlet gas was pre-heated and mixed with steam in the vaporizer. The steam was carried into the reactor via the inlet gas. The tubes between the vaporizer and reactor were wrapped with heating tape to maintain a constant temperature of 423 K.

### 2.3. Flue Gas Analysis

The exhaust flue gas was first passed through a filter to separate the fly ash, the water was removed from the condenser, and finally, it was dried before entering the analyzers. The gas composition of flue gas (including NO, SO_2_, O_2_, CO, and CO_2_) concentrations were measured with a KM9106 gas analyzer (Kane International Limited, Welwyn Garden City, UK). The NO was measured with an electrochemical sensor. The gas analyzer was calibrated before the combustion tests.

### 2.4. Combustion Conditions

The combustion tests were conducted in either O_2_/H_2_O or O_2_/N_2_ mixtures. During all experiments, the feed rate of the coal samples was kept constant at 0.3 g/min, and the ratio of the primary to secondary streams was one-fourth. Comparative experiments were carried out with different parameters to investigate the influences of the atmosphere, oxygen concentration, temperature, and excess oxygen ratio on the NO emissions. The combustion conditions are summarized in Table 3. The excess oxygen ratio was defined as follows:

(1) $$\lambda =\frac{{\left(m_{O_{2}}/m_{\mathrm{fuel}}\right)}_{\mathrm{actual}}}{{\left(m_{O_{2}}/m_{\mathrm{fuel}}\right)}_{\mathrm{stoichiometric}}}$$

The combustion experiment’s procedure is described as follows:

1. Check the airtightness of the combustion system before all tests.
2. Heat the reactor to the experimental temperature and adjust the gas flow rate to the corresponding value according to the combustion conditions.
3. Open the gas analyzer and monitor the flue gas composition in the reactor.
4. Start the micro-feeding device when the reaction gas composition reaches the designed experimental condition.

The emission concentrations were recorded after the steady-state and steady-flow conditions were reached.

Emission values must consider varying fuel mass inputs for a more accurate comparison of these combustion cases. Therefore, fuel-based emissions (mg/g coal feed) were used to capture the differences in the fuel mass input. The formula for calculating these fuel-based emissions was as follows:

(2) $$X_{NO}=\frac{C_{NO}\times Q}{B}$$

where $X_{NO}$ is the fuel-based NO concentration (mg NO/g coal feed), $C_{NO}$ is the NO concentration measured in the flue gas (mg/m^3^), Q is the total flow rate of the flue gas converted to the condition of 1 atm and 273 K from the rate obtained with a wet gas meter (L/min) at the reactor outlet, and B is the fuel feed rate (g/min).

## 3. Results and Discussion

### 3.1. Effect of Atmosphere on NO Emissions

**Figure 2:**
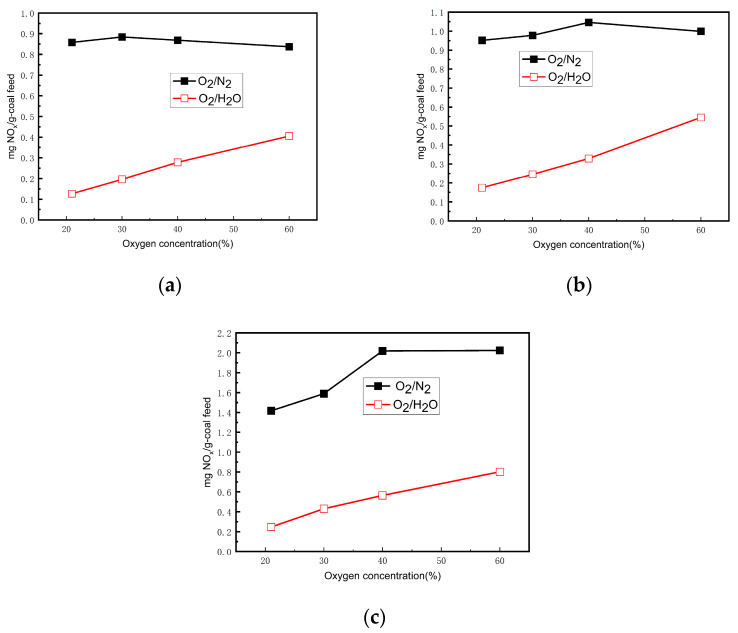
NO concentrations of SF pulverized coal under different atmospheres. (**a**) 1173 K, (**b**) 1273 K, (**c**) 1373 K.

The Shen Fu coal was burned in O_2_/H_2_O and O_2_/N_2_ atmospheres at different temperatures. The NO concentrations (mg NO/g coal feed) are shown in Figure 2. Figure 2 demonstrates that the NO concentrations of SF coal combustion in the O_2_/H_2_O atmosphere were much lower than those in the O_2_/N_2_ atmosphere. Compared with the O_2_/N_2_ atmosphere, N_2_ was absent in the O_2_/H_2_O atmosphere; as such, thermal NO and prompt NO are absent during oxy-steam combustion. The temperature in the experiments was below 1373 K; thus, the proportion of thermal NO and prompt NO in the total NO was very low in the O_2_/N_2_ atmosphere. Consequently, the absence of N_2_ during oxy-steam combustion is not the main reason for the much lower NO concentration in the O_2_/H_2_O atmosphere.

The effect of steam on the conversation of fuel-N should be figured out to analyze the reason for the decreasing NO concentration in the O_2_/H_2_O atmosphere. The fuel-N was mainly transformed into volatile-N (NH_3_, HCN, and HCNO) and char-N during the coal devolatilization process. According to Tian’s study [22], when steam is present in the reactant gas, NH_3_ is the primary volatile-N product during the coal devolatilization process due to the amount of H produced from H_2_O, and the amount of volatile-N is larger than that in the corresponding O_2_/N_2_ atmosphere. Moreover, the hydrolysis reaction of HCN (R1) is enhanced in a high-steam-concentration atmosphere with a temperature exceeding 650 °C, resulting in an amount of HCN being converted to NH_3_ [36]. Consequently, NH_3_ is the predominant product of volatile-N during oxy-steam combustion.

HCN + H_2_O → NH_3_ + CO (R1)

Meanwhile, the char-N is transformed into a gaseous nitrogen-containing compound via a set of heterogeneous reactions. Park found that HCN was the primary product of the reaction of char-N with H_2_O, and the amount of available hydrogen greatly affected the formation rate of HCN [26,27]. The studies by Chang and McKenzie showed that the NH_3_ yield was much higher when steam was added to the atmosphere during coal gasification, and the HCN was also slightly higher [21,25]. They claimed that steam was vital in converting coal/char-N into NH_3_ by providing H on the char surface. Consequently, the proportion of char-N converted into NH_3_ and HCN should be much higher in an O_2_/H_2_O atmosphere than in an O_2_/N_2_ atmosphere with an identical O_2_ concentration.

In summary, the absence of steam promotes fuel-N to transform into NH_3_ and HCN in an O_2_/H_2_O atmosphere. Steam can also affect the conversion of NH_3_ and HCN. According to the previous studies by Yue [29] and He [37], the concentration of OH is higher in an O_2_/H_2_O atmosphere than in an O_2_/N_2_ atmosphere due to the enhancement of R2 and R3 in a high-steam concentration atmosphere [38].

2OH → O + H_2_O (R2)

H + H_2_O → OH + H_2_ (R3)

The conversion reaction (R4 and R5) of HCN to NO is enhanced due to the high concentration of OH in an O_2_/H_2_O atmosphere.

HNO + OH → NO + H_2_O (R4)

NH + H_2_O → HNO + H_2_ (R5)

The rate of product of R5 drastically increases due to the chaperone effect of H_2_O. HCN→NCO→HNCO→NH_2_→NH→HNO→NO becomes the main pathway of NO formation from HCN in an O_2_/H_2_O atmosphere.

In addition, the conversion rate of NH_3_ to NO in O_2_/H_2_O atmospheres compared with O_2_/N_2_ atmospheres is enhanced due to the high concentration of H_2_O, and the pathway of NH_3_→NH_2_→NH→HNO→NO is predominant in O_2_/H_2_O atmospheres.

Consequently, the conversion rates of HCN and NH_3_ to NO in O_2_/H_2_O atmospheres are higher than in O_2_/N_2_ atmospheres. Thus, the NO formation from the oxidation of fuel-N is enhanced in an O_2_/H_2_O atmosphere. Two opposed mechanisms affect the NO concentration during coal combustion: the NO formation and reduction mechanisms. Consequently, the NO emissions largely depend on the competition between the NO formation and destruction mechanisms.

Based on this analysis, a high steam concentration is beneficial to the large proportion of fuel-N converted to NH_3_ and HCN and of NH_3_ and HCN converted to NO in O_2_/H_2_O atmospheres. The NO emissions, however, are much lower in an O_2_/H_2_O atmosphere than in an O_2_/N_2_ atmosphere with an identical O_2_ concentration, as shown in Figure 2. This means that a large amount of NO is decomposed during coal combustion in an O_2_/H_2_O atmosphere via the NO destruction reaction.

One reason for the decomposition of NO is the selective non-catalytic reaction (SNCR) mechanism [39,40]:

4NH_3_ + 4NO + O_2_ → 4N_2_ + 6H_2_O (R6)

Because of the large amount of NH_3_ formation in an O_2_/H_2_O atmosphere, some percentage of the NH_3_ becomes a reductant at temperatures of 1073–1373 K.

Another reason is that the char plays an important role as a catalyst for the destruction of NO, either directly or by reacting with CO or H_2_ [41].

2NO + 2CO → N_2_ + 2CO_2_ (R7)

2NO + 2H_2_ → N_2_ + 2H_2_O (R8)

In an O_2_/H_2_O atmosphere, steam gasification reaction R9 produces a large amount of CO, and the steam shift reaction R10 produces a large amount of H_2_; thus, the concentrations of H_2_ and CO are much higher than those in an O_2_/N_2_ atmosphere. Consequently, a significant amount of NO is decomposed in an O_2_/H_2_O atmosphere.

C + H_2_O → CO + H (R9)

CO + H_2_O → H_2_ + CO_2_ (R10)

### 3.2. Effect of Oxygen Concentration on NO Emissions

**Figure 3:**
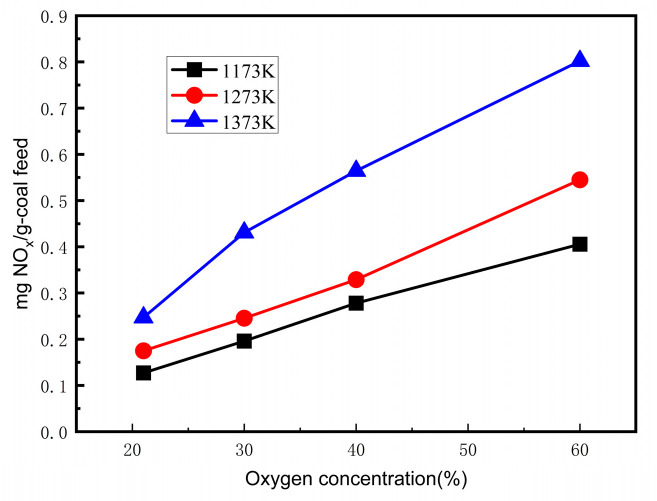
NO concentration versus oxygen concentration at different temperatures in O_2_/H_2_O atmosphere.

Figure 3 shows the NO emissions versus oxygen concentration at different temperatures in an O_2_/H_2_O atmosphere. In the oxygen concentration range of 21 vol.%–60 vol.%, the NO concentration increased with the increasing oxygen concentration, and the increase in the NO emissions at a temperature of 1373 K was faster than that at 1173 and 1273 K. The oxidizing atmosphere gradually strengthened with the increasing oxygen concentration in the reactant, resulting in the oxidation of NH_3_ and HCN into NO. Meanwhile, the reducing atmosphere weakened as the oxygen concentration increased; thus, the decomposition amount of NO decreased. In addition, at a lower steam concentration, the gas residence time was increased at the lower total flow rate (at 1173 K, 1273 K, and 1373 K). The increased oxygen concentration resulted in a higher flame temperature. Thus, the conversion of fuel-N into NO was promoted due to the longer residence time and higher flame temperature, and the NO concentration was higher with the higher oxygen concentration.

### 3.3. Effect of Temperature on NO Emissions

**Figure 4:**
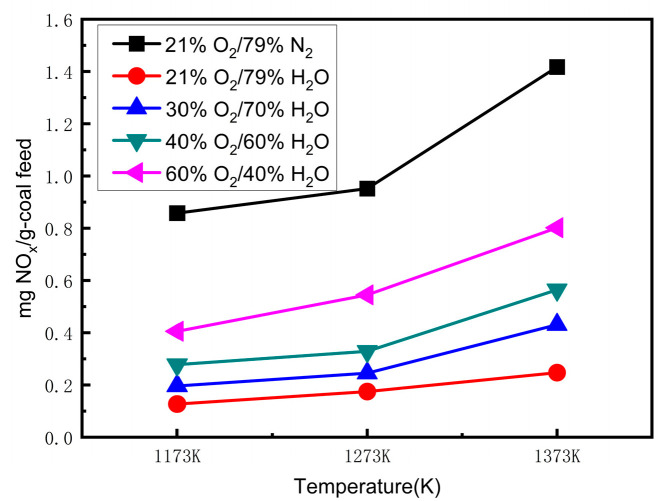
NO concentration versus temperature at different oxygen concentrations.

The NO emissions at different temperatures with an oxygen concentration varying from 21% to 60% in O_2_/H_2_O mixtures are shown in Figure 4. Figure 4 demonstrates that the NO emissions increased as the temperature increased in the O_2_/H_2_O atmosphere. The NO emissions increased by almost two times from 1173 K to 1373 K in the O_2_/H_2_O atmosphere. The transformation and oxidation of fuel-N were accelerated with the increasing combustion temperature. Furthermore, at high temperatures, the water vapor diluted the reductive gas atmosphere and shortened the gas residence time in the reactor to weaken the reduction of NO. Therefore, the NO emissions increased. Even in the 60% O_2_/40% H_2_O atmosphere, the NO emissions were still much lower than those in the 21% O_2_/79% N_2_ (air) atmosphere at the temperatures of 1173 K, 1273 K, and 1373 K. Hence, a remarkable amount of NO was reduced in the O_2_/H_2_O atmosphere. The reduction of NO plays an important role in the transformation of fuel-N in an O_2_/H_2_O atmosphere.

### 3.4. Effect of Excess Oxygen Ratio on NO Emissions

**Figure 5:**
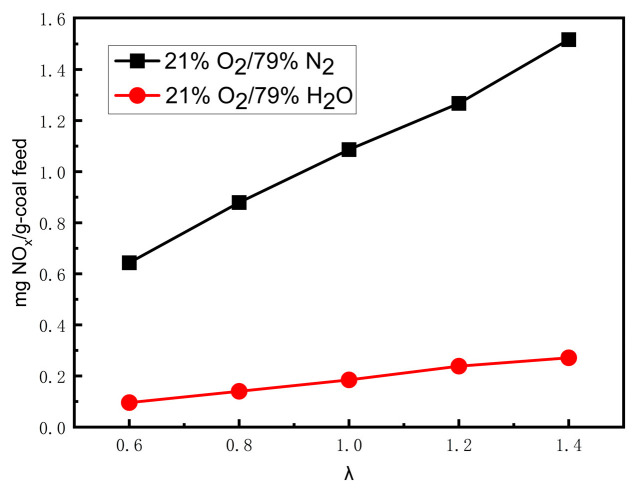
NO concentration versus excess oxygen ratio under different atmospheres (21% O_2_).

The effect of the excess oxygen ratio (λ) on the NO emissions was investigated in this study. As the coal feed rate was kept constant, λ was varied by changing the total reaction gas flow rate. Figure 5 shows the NO emissions at different excess oxygen ratios in 21% O_2_/79% H_2_O and 21% O_2_/79% N_2_. Figure 5 indicates that the NO concentration monotonically and significantly increased with the increasing λ in the O_2_/N_2_ atmosphere, while it slightly increased in the O_2_/H_2_O atmosphere. This may be because although the oxidation of fuel-N increased with the increase in the O_2_ amount, the reduction of NO in the O_2_/H_2_O atmosphere also increased with the increase in the H_2_O amount.

## 4. Conclusions

The NO formation characteristics during the combustion of Shen Fu coal in O_2_/H_2_O and O_2_/N_2_ mixtures were investigated experimentally in a drop-tube furnace.

The NO concentrations (mg NO/g coal feed) were compared between 21% O_2_/79% H_2_O and 21% O_2_/79% N_2_ atmospheres at 1173 K, 1273 K, and 1373 K. The results show that the NO concentrations of SF coal combustion in the 21% O_2_/79% H_2_O atmosphere were much lower than those in the 21% O_2_/79% N_2_ atmosphere at these three temperatures. Although water vapor is conducive to NO formation, the NO reduction reaction is dominant during the coal combustion process in an O_2_/H_2_O atmosphere. The reasons for the decomposition of NO include the selective non-catalytic reaction (SNCR) mechanism and the important role that char plays as a catalyst for the destruction of NO, either directly or by reacting with CO or H_2_.

The NO concentrations significantly increased with the increasing oxygen concentration. High oxygen concentrations create an oxidizing environment, which enhances the NO formation reaction. In addition, a longer residence time and higher flame temperature also contribute to the NO increase at a higher oxygen concentration. NO concentration increased with the increasing temperature in both the O_2_/H_2_O and O_2_/N_2_ conditions. This was caused by the acceleration of the fuel-N oxidation reaction. Moreover, the reduction reaction of NO was weakened due to the shortened residence time. The results show that the excess oxygen ratio (λ) slightly impacts the NO emissions in an O_2_/H_2_O atmosphere.

The results obtained can provide a better understanding of NO emission mechanism of coal combustion in O_2_/H_2_O atmosphere. Future work is planned on different coals with lower nitrogen content.

## Figures and Tables

**Table 1:** Physical properties of H_2_O, CO_2_, and N_2_ (1000 K; 0.1 MPa).

Parameter	H_2_O	CO_2_	N_2_	$\frac{H_{2}O}{N_{2}}$	$\frac{H_{2}O}{{CO}_{2}}$
Density (kg/m^3^)	0.22	0.54	0.34	0.64	0.41
Heat capacity (J/mol K)	41.29	54.32	32.71	1.26	0.76
Heat conductivity (W/m K)	0.097	0.071	0.066	1.47	1.38
Dynamic viscosity (Pa s)	3.76 × 10^−5^	4.13 × 10^−5^	4.16 × 10^−5^	0.9	0.91
Thermal diffusivity (m^2^/s)	1.95 × 10^−4^	1.08 × 10^−4^	1.68 × 10^−4^	1.16	1.81
Mass diffuse coefficient (m^2^/s)	1.27 × 10^−4^	8.25 × 10^−5^	9.48 × 10^−5^	1.34	1.54

**Table 2:** Properties of coal samples.

Proximate Analysis (wt%, ad)				Ultimate Analysis (wt%, ad)					Q_net,ar_ (MJ/kg)
Moisture	Volatile Matter	Ash	Fixed Carbon	C	H	O (By Difference)	N	S	23.15
6.43	36.95	2.86	53.76	66.05	4.43	19.01	0.84	0.38	

**Table 3:** Operation conditions for combustion experiments.

Mode	Temperature (K)	O_2_ Concentration (vol. %)	Total Volume Flow Rate (STP, L/min)	λ
O_2_/H_2_O O_2_/N_2_	1173 K 1273 K 1373 K	21 30 40 60	1.6	0.83
				1.19
				1.58
				2.38
O_2_/N_2_	1373 K	21	1.15	0.6
			1.54	0.8
			1.92	1
			2.31	1.2
			2.69	1.4
O_2_/H_2_O	1373 K	21	1.37	0.6
			2.06	0.8
			2.56	1
			3.08	1.2
			3.62	1.4

## Data Availability

The original contributions presented in this study are included in this article. Further inquiries can be directed to the corresponding author.
