# Correction: Zhang et al. NO Emission Characteristics of Pulverized Coal Combustion in O_2_/N_2_ and O_2_/H_2_O Atmospheres in a Drop-Tube Furnace. *Materials* 2024, *17*, 4997

**DOI:** 10.3390/ma19030517

**Published:** 2026-01-28

**Authors:** Liang Zhang, Jun Fan, Changlin Wang, Jiaqi Yuan, Cen Hao, Shiying Cao

**Affiliations:** 1 School of Information Engineering, Jiangsu Open University, Nanjing 210036, China; zhangliang@jsou.edu.cn (L.Z.); fanjun@jsou.edu.cn (J.F.); wangcl@jsou.edu.cn (C.W.); nayuanjiaqi@126.com (J.Y.); haocen@jsou.edu.cn (C.H.); 2 School of Mechanical and Electrical Engineering, Shenzhen Polytechnic University, Shenzhen 518055, China

In the original publication [1], there was an overlap in Figure 2. The corrected Figure 2 appears below. The authors state that the scientific conclusions are unaffected. This correction was approved by the Academic Editor. The original publication has also been updated.

## Figures and Tables

**Figure 2:**
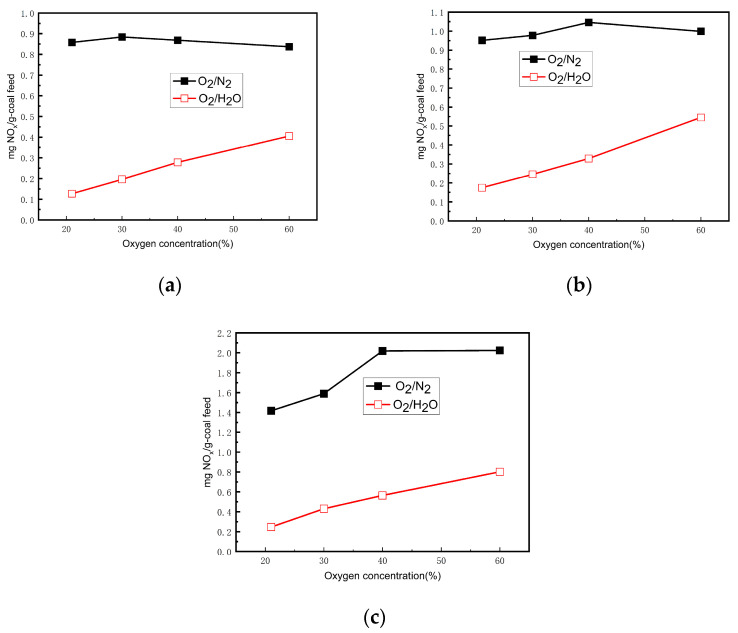
NO concentrations of SF pulverized coal under different atmospheres. (**a**) 1173 K, (**b**) 1273 K, (**c**) 1373 K.
